# Is Recent Exposure to Antibiotics a Risk Factor for Hospitalisation in Korean Children with Acute Non-Bacterial Gastroenteritis? A Nationwide Population-Based Study

**DOI:** 10.3390/children8090809

**Published:** 2021-09-15

**Authors:** Dongbum Suh, Hyuksool Kwon

**Affiliations:** 1 Department of Emergency Medicine, Seoul National University Bundang Hospital, Seongnam-si 13620, Korea; dongbum@snubh.org; 2 Department of Emergency Medicine, Seoul National University College of Medicine, Seoul 03080, Korea

**Keywords:** antibiotics, acute gastroenteritis, hospitalization, probiotics, risk factor

## Abstract

The purpose of this study was to evaluate the effect of recent antibiotic therapy and probiotics on hospitalisation in children with acute gastroenteritis. Using a retrospective study design, data from the population aged up to 18 years were collected from the Korean National Health Insurance Service-National Sample Cohort. The duration of antibiotic therapy within 14 days of the index visit, prescription of probiotics at initial presentation, the effect size of antibiotic exposure on hospitalisation, and its modification by probiotics were assessed. Of 275,395 patients with acute gastroenteritis, 51,008 (18.5%) had prior exposure to antibiotics. Hospitalisation within 7 days of the index visit was positively associated with exposure to antibiotics (*p*-trend < 0.001). The prescription of probiotics (as a main effect; odds ratio, 0.80; 95% confidence interval 0.72–0.87) was associated with a decreased risk of hospitalisation. Prior exposure to antibiotics might be a significant risk factor for hospitalisation in children presenting with acute gastroenteritis. This may be favourably modified by administering probiotics at the initial presentation.

## 1. Introduction

Acute gastroenteritis (AGE) is a very common illness in children, with almost 1.7 million children in the US seeking medical care for this condition every year [1]. Although it is commonly of viral origin and is usually self-limited, it represents a significant socioeconomic burden because of its high incidence and because it can culminate in unwanted hospitalisations [2].

The ecological environment of the intestine is maintained by a delicate balance of more than 400 species of bacteria, which are called the gut microbiota [3]. The endogenous gut microbiota plays critical roles in maintaining gastrointestinal integrity by protecting against epithelial cell injury, regulating host fat storage, and stimulating intestinal angiogenesis [4,5,6,7]. However, exposure to antibiotic treatments can disrupt the delicate balance of gut microbiota by causing an intestinal overgrowth of pathogenic microorganisms [8].

Any recent exposure to antibiotics might influence the susceptibility of the host to AGE and its complications. An epidemiological study conducted at a single regional centre reported that recent antibiotic therapy in children increased the probability and severity of AGE [9]. However, this study may have been biased due to the small study population and inaccurate history of antibiotic use.

Probiotics refer to preparations of non-pathogenic live microorganisms that have a health benefit for the host [10,11]. Probiotics are known to improve the balance of gut microbiota in humans [12] and have been tried for the treatment of AGE and antibiotic-associated diarrhoea, but their effectiveness is still controversial [13,14,15,16].

In this study, we conducted a large-scale, population-based observational study using a nationally representative cohort to prove the hypotheses that (1) recent exposure to antibiotics would have a negative impact on patients with AGE and (2) this negative impact could be significantly reduced by prescribing probiotics at the time of initial presentation.

## 2. Materials and Methods

### 2.1. Study Design, Setting, and Ethical Approval

This was a retrospective cohort study that used a nationwide population-based health insurance dataset. The data source was the National Health Insurance Service (NHIS)—National Sample Cohort (NHIS–NSC), a population-based cohort established by the Korean NHIS. All Korean citizens have been obligatorily registered with the NHIS. This study was approved by the Institutional Review Board of Seoul National University Bundang Hospital (IRB No. X-1607/356-905). Patient consent was waived due to the retrospective nature of the study and the analysis of anonymous clinical data.

### 2.2. Data Source

The NHIS–NSC contains the claims data of one million individuals who were randomly sampled from the entire Korean population after stratification, representing 2% of health insurance beneficiaries. It provides diagnostic codes based on the International Classification of Diseases (ICD)-10 coding system, prescription codes based on the Anatomical Therapeutic Chemical (ATC) classification system, procedure codes and related costs, and patient characteristics such as age, sex, and socioeconomic status. By combining these data, it is possible to know patients’ medication history and whether or not they were hospitalised in a general ward or in the intensive care unit. It also includes information about disability and death based on data from the National Disability Registry and death certificates, respectively. We used the most recent release, which contained claims data from 2002 to 2013. A detailed description of the cohort can be found in a previous paper [17].

### 2.3. Target Population, Term Definitions, and Outcome Measures

The target population included paediatric patients aged up to 18 years and registered from 2002 to 2013. The inclusion criterion was a new case of presumed non-bacterial AGE, defined as a new visit with a primary diagnosis of viral or non-specific gastroenteritis (A08.x and A09.x, respectively) and without the prescription of systemic antibiotics (ATC code J01) or hospitalisation on the same day. We excluded patients with any previous visit for any gastroenteritis or gastrointestinal symptoms in the past month, as determined by the presence of the ICD-10 diagnostic codes A00.x-A09.x or R10-R19.x in the primary diagnosis, so as to only select cases with AGE. We also excluded patients who had been hospitalised within three months prior to the index visit, to ensure that relatively healthy children were selected.

Exposure was defined as the duration of antibiotic therapy within 14 days of the index visit. To avoid miscalculating the number of days of antibiotic use due to overlapping prescriptions in separate clinics or hospitals, the date and duration on each prescription were reviewed and modified to obtain the actual date of antibiotic use. We categorised the exposure into four categories: no exposure and exposure for 1–3 days, 4–7 days, and more than 7 days. Prescription of probiotics (A07F) at the time of the index visit was included as a covariate. To analyse the indication for recent antibiotic therapy, we retrieved the ICD-10 codes and grouped them as follows: (1) Respiratory tract infections (J00-J99) for nasopharyngitis (common cold), sinusitis, pharyngitis, tonsillitis, laryngitis and tracheitis, croup, epiglottitis, pneumonia, bronchitis, bronchiolitis, and peritonsillar abscess; (2) genitourinary (N00-N99) for urinary tract infection, epididymo-orchitis, vulvovaginitis, cervicitis, and pelvic inflammatory disease; (3) skin, bone, and soft tissue (L00-M99) for infectious arthropathies, osteomyelitis, cellulitis, impetigo, and infectious dermatitis; (4) external and middle ear (H60-H95) for otitis media, otitis externa, middle ear effusion, and mastoiditis; and (5) central nervous system (A80-A89) for meningitis and encephalitis. Patient age was categorised as follows: <1 year, 1–6 years, 7–12 years, and 13–18 years.

The primary outcome was hospitalisation within seven days of the index visit, and secondary outcomes were intensive care unit (ICU) admission and death during hospitalisation.

### 2.4. Study Objectives

The primary objective of this study was to determine whether prior exposure to antibiotics at the time of initial presentation was a significant risk factor for subsequent hospitalisation in children with AGE. The secondary objective was to determine whether the prescription of probiotics at the initial presentation could significantly modify the increased risk in a favourable way.

### 2.5. Statistical Analysis

Comparisons between groups were made using the chi-squared test or Fisher’s exact test, as appropriate. Logistic regression was used to assess the linear association between binary and ordinal variables. Spearman’s test was used to assess the correlation between two ordinal variables. We first stratified all cases according to the days of antibiotic exposure and assessed the crude risk of hospitalisation within each stratum. We then constructed a multivariable logistic regression model with an interaction term between antibiotic exposure and probiotic prescription, to adjust for confounders and test whether the prescription of probiotics significantly modified the effect of prior antibiotic exposure. The results of the logistic regression analyses are presented as odds ratios (ORs) and their 95% confidence intervals (CIs). Statistical significance was set at *p* < 0.05. All data handling and statistical analyses were performed using R-packages version 3.3.2 (R Foundation for Statistical Computing, Vienna, Austria). 

## 3. Results

In total, 275,395 AGE cases were identified. Among them, 51,008 (18.5%) patients had prior exposure to antibiotics (Table 1).

In all exposure groups, the proportion of boys was higher than that of girls, and the most common age group was 1–6 years. The most common type of recent infection was respiratory infection, with an overall admission rate of 1.3%. Every type of recent infection was positively associated with recent antibiotic exposure (all *p*-trends < 0.001). Hospitalisation, as the primary outcome, was significantly different among the groups of different antibiotic exposure durations and was positively associated with antibiotic exposure (*p* < 0.001 and *p*-trend < 0.001). However, the secondary outcomes were not significantly associated with antibiotic exposure (*p* = 0.563 and *p*-trend = 0.255 for ICU admission; *p* = 0.796 and *p*-trend = 0.421 for death).

The whole study sample was stratified into two strata, with and without probiotic prescription, and the association between antibiotic exposure and hospitalisation risk was assessed (Table 2). The number of days prior to antibiotic exposure was positively associated with subsequent hospitalisation, irrespective of probiotic use. The effect size of antibiotic exposure was significantly lower when probiotics were prescribed in cases with more than 3 days of exposure (*p* = 0.023 for 4–7 days of exposure, *p* < 0.001 for >7 days).

We constructed a multivariable logistic regression model with an interaction term between antibiotic exposure and the prescription of probiotics (Table 3). An older age and the prescription of probiotics (as a main effect; OR 0.80; 95% CI 0.72–0.87) were associated with a decreased risk of hospitalisation, while exposure to antibiotics and a recent history of respiratory and CNS infections were associated with increased risk. The interaction term was statistically significant when the antibiotic exposure was prolonged (>7 days, OR 0.53; 95% CI, 0.43–0.83; *p* = 0.002). 

Using a linear combination, we estimated the effect size of recent antibiotic exposure in patients with and without probiotic prescriptions (Table 4). In the case of exposure to antibiotics for more than 7 days, the effect size was only significantly lower when probiotics were also prescribed (*p* = 0.002 for >7 days of exposure).

## 4. Discussion

There are few epidemiologic studies that have investigated the association between antibiotics and enteritis. We believe that our study is the first to describe an association between antibiotic use and enteritis using data from a national cohort. We found that antibiotic exposure was a significant risk factor for subsequent hospitalisation in Korean children with AGE, and that this increased risk could be modified by the prescription of probiotics at the initial presentation. The findings of the present study are consistent with a previous study that reported an association between previous antibiotic use in children and an increased rate of AGE, regardless of aetiology. The reported association was stronger with recent antibiotic use [9]. However, to our knowledge, our finding regarding the interplay between prior antibiotic exposure and probiotic prescription in children with AGE is novel and suggests that it may be important to maintain the correct balance of gut microbiota in children with AGE.

In our study, we did not clarify the reason for the association between a longer duration of antibiotic exposure and a higher rate of hospitalisation. However, we believe that the ecological disturbance of the gut microbiota caused by a longer period of antibiotic exposure may reduce intestinal motility and result in the proliferation of pathogenic organisms, leading to persistent AGE. Importantly, probiotic supplementation may reduce this malicious process. Our study showed that probiotics were associated with a decreased risk of hospitalisation for AGE after long-term antibiotic use. This effect of probiotics has not been fully evaluated and might be mediated by numerous factors, including the production of antimicrobial substances, local competition for adhesion receptors and nutrients, and stimulation of intestinal antigen-specific and nonspecific immune responses, especially with longer periods of antibiotic exposure [18,19].

Table 3 shows that older children may have lesser odds of hospitalisation for AGE, even if they were exposed to antibiotics. This finding suggests that children younger than 6 years of age were more susceptible to dehydration; even in cases of relatively mild AGE, oral ingestion became difficult, and hospitalisation was required more often. This result is consistent with that of a previous study [2].

This study has several limitations. First, we analysed data from more than seven years ago, and it is a concern that this cohort might not accurately reflect the current practice for acute gastroenteritis. However, there is no change in the fact that antibiotics can change the gut microbiota, and probiotics are still prescribed in current practice to reduce this change. Therefore, it is expected that the results of this study are applicable to the current environment. Second, the severity of disease and relevant laboratory data are not reported in the NHIS–NSC; thus, we could not precisely determine the appropriateness of the treatment of children with AGE. As an alternative, we defined hospitalisation as a severity marker of AGE because most AGE cases are self-limited, do not require hospitalisation, and have a very good prognosis with proper management in developed countries. Third, because of the limitations of the NHIS–NSC dataset, we only analysed the diagnostic codes that matched nonspecific and viral AGE. As a result, we excluded bacterial AGE codes, and thus, we could not assess the association between bacterial AGE and probiotic prescription. Individualised studies on bacterial AGE would be a good option to address this limitation. Fourth, changes in the human gut microbiota vary depending on the type of antibiotic and probiotics used, but our data did not include the kind of antibiotics and probiotics. We could not define which antibiotics are harmful and which probiotics are protective. However, we tried to elucidate the overall effects of antibiotics and probiotics and raise the cautions about prescriptions without notice. In addition, although the association between the period of antibiotic prescription and hospitalisation patterns among children with AGE was evident in our results, given the nature of cohort studies, we cannot confirm whether antibiotics are a true risk factor or are simply associated with hospitalisation. However, we aimed to determine whether antibiotics could be a factor affecting hospitalisation. Further studies should be conducted to specifically address antibiotic exposure as a leading cause of hospitalisation. Finally, although we adjusted extensively for possible comorbidities, unmeasured confounders remain an issue. Given the nature of our dataset, we could not adjust for some important risk factors, such as body mass index, diet, and family history.

## 5. Conclusions

In conclusion, prior exposure to antibiotics is a significant risk factor for subsequent hospitalisation in paediatric patients with AGE, and this increased risk can be modified favourably by the prescription of probiotics at the initial presentation. Considering the beneficial effect of probiotics for a prior history of antibiotic exposure, it would be desirable to prescribe probiotics for any child with AGE, especially when the patient has a recent history of antibiotic therapy within 14 days of the visit.

## Figures and Tables

**Table 1:** Baseline characteristics and outcome events.

Exposure Groups	Duration of Antibiotics Exposure				*p*-Value	*p*-Trend
	No Exposure (N = 224,387)	1–3 Days (N = 30,463)	4–7 Days (N=15,354)	>7 Days (N = 5191)		
Age					<0.001	<0.001
<1 year	44,663 (19.9)	6155 (20.2)	3335 (21.7)	1315 (25.3)		
1–6 years	71,128 (31.7)	14,703 (48.3)	8542 (55.6)	3082 (59.4)		
7–12 years	65,642 (29.3)	6555 (21.5)	2530 (16.5)	595 (11.5)		
>13 years	42,954 (19.1)	3050 (10.0)	947 (6.2)	199 (3.8)		
Sex					<0.001	<0.001
Boys	118,805 (52.9)	16,711 (54.9)	8464 (55.1)	2927 (56.4)		
Site of recent infection *						
Respiratory	22,589 (10.1)	23,674 (77.7)	13,473 (87.7)	4759 (91.7)	<0.001	<0.001
Genitourinary	296 (0.1)	338 (1.1)	183 (1.2)	87 (1.7)	<0.001	<0.001
Skin, bone, and soft tissue	2191 (1.0)	1634 (5.4)	1075 (7.0)	338 (6.5)	<0.001	<0.001
External and middle ear	580 (0.3)	2425 (8.0)	2898 (18.9)	1889 (36.4)	<0.001	<0.001
Central nervous system	35 (0.02)	25 (0.1)	12 (0.1)	3 (0.1)	<0.001	<0.001
Prescription of probiotics	145,809 (65.0)	20,722 (68.0)	10,896 (71.0)	3747 (72.2)	<0.001	<0.001
Outcomes						
Hospitalisation	2634 (1.2)	607 (2.0)	310 (2.0)	151 (2.9)	<0.001	<0.001
ICU admission	6 (0.003)	2 (0.007)	0 (0.000)	0 (0.000)	0.563	0.255
Death	3 (0.001)	1 (0.003)	0 (0.000)	0 (0.000)	0.796	0.421

The values are expressed as number (%). * Since each site of infection was counted in the case of co-infection, the total of the proportion of sites reported may exceed 100%.

**Table 2:** Crude association between antibiotic exposure and hospitalisation, with and without probiotics, and their differences in multiplicative scale.

Duration of Antibiotic Exposure	All Cases	Without Probiotics	With Probiotics	Difference	*p* Value
1–3 days	1.71 (1.57–1.87)	1.87 (1.65–2.12)	1.65 (1.45–1.87)	0.88 (0.75–1.03)	0.123
4–7 days	1.73 (1.54–1.95)	2.09 (1.77–2.47)	1.60 (1.35–1.89)	0.77 (0.62–0.96)	0.023
>7 days	2.52 (2.14–2.98)	3.52 (2.80–4.42)	2.06 (1.61–2.64)	0.59 (0.53–0.65)	<0.001

The values are expressed as odds ratio (95% confidence interval).

**Table 3:** Multivariable logistic regression model of hospitalisation within a week.

Variables	OR *	*p*-Value
Age		
<1 year	1 (referent)	
1–6 years	0.88 (0.81–0.95)	<0.001
7–12 years	0.46 (0.41–0.50)	<0.001
>13 years	0.54 (0.46–0.63)	<0.001
Sex Girls		
	0.96 (0.90–1.03)	0.102
Site of recent infection		
Respiratory	1.51 (1.32–1.71)	<0.001
Genitourinary	1.50 (1.00–2.24)	0.051
Skin, bone, and soft tissue	0.99 (0.83–1.17)	0.680
External and middle ear	1 (referent)	
Central nervous system	4.24 (1.68–10.55)	<0.001
Duration of antibiotics exposure		
No exposure	1 (referent)	
1–3 days	1.24 (1.05–1.49)	0.044
4–7 days	1.24 (1.06–1.60)	0.040
>7 days	2.05 (1.59–2.83)	<0.001
Probiotics prescription	0.80 (0.72–0.87)	<0.001
Interaction terms		
Exposure 1–3 days * probiotics	0.92 (0.76–1.14)	0.511
Exposure 4–7 days * probiotics	0.81 (0.64–1.00)	0.052
Exposure >7 days * probiotics	0.53 (0.43–0.83)	0.002

The values are expressed as odds ratio (95% confidence interval). * Adjusted for age, sex, socioeconomic status, infection sites, antibiotic exposure duration, and probiotic prescription. OR: odds ratio.

**Table 4:** Adjusted odds ratios * for hospitalisation, with and without probiotics, and their difference in a multiplicative scale.

Duration of Antibiotic Exposure	Without Probiotics	With Probiotics	Difference	*p*-Value
1–3 days	1.28 (1.10–1.52)	1.18 (1.02–1.34)	0.92 (0.76–1.14)	0.511
4–7 days	1.35 (1.10–1.69)	1.10 (0.89–1.31)	0.81 (0.64–1.00)	0.052
>7 days	2.43 (1.84–3.00)	1.29 (1.10–1.57)	0.53 (0.43–0.83)	0.002

The values are expressed as odds ratio (95% confidence interval). * Adjusted for age, sex, socioeconomic status, and infection sites.

## Data Availability

Data were obtained from the National Health Insurance Sharing Service (NHISS) on the NHIS database and are available online https://nhiss.nhis.or.kr/bd/ab/bdaba021eng.do (accessed on 12 September 2021) with the permission of the NHIS.
